# Risk of periprosthetic femur fracture after anterior cortical bone windowing: A mechanical analysis of short versus long cemented stems in pigs

**DOI:** 10.3109/17453674.2011.636670

**Published:** 2011-11-25

**Authors:** Lance J Wilson, Corey J Richards, Dean Irvine, Armand Tillie, Ross W Crawford

**Affiliations:** Institute of Health and Biomedical Innovation, Queensland University of Technology, Brisbane, Australia

## Abstract

**Background and purpose:**

Removal of distal cement at femoral implant revision is technically challenging and is associated with complications such as cortical perforations. A technique that can reduce the risks and operating time is to make a small cortical window in the distal femur for enhanced access. We wanted to determine whether the use of long, bridging, cemented femoral stems is necessary to reduce the risk of postoperative periprosthetic fractures after using an anterior cortical bone window.

**Methods:**

66 fresh pig femurs underwent mechanical testing. Steel rods were implanted at 3 locations: (1) at the distal window edge, (2) 15 mm proximally to the cortical window edge, and (3) 15 mm distally. 54 femurs were tested using a 3-point bending setup and 12 femurs were tested using a torsional load setup.

**Results:**

Load to fracture ratio and bending stiffness ratio were similar in the 3 groups, for either the 3-point bending test or the torsional load test.

**Interpretation:**

Our findings suggest that bypass of cortical windows with a revision femoral component may not reduce the risk of periprosthetic fracture.

The removal of well-fixed cement is difficult and time consuming. Various surgical techniques and instruments have been developed to facilitate cement removal: extended trochanteric osteotomy (Paprosky et al. 2001), cortical windows (Nelson and Weber 1981), cement removal osteotomes/gauges/reamers (Gray 1992), and ultrasound probes (Goldberg et al. 2007). Iatrogenic femoral host bone loss, inadvertent perforation, and femoral fracture are the main risks associated with the removal of cement (Klein and Rubash 1993).

The use of cortical windows, as initially described by Nelson and Weber (1981), reduces the risk of perforation at the revision surgery, while allowing for full weight bearing. The window is typically made near the tip of the implant to facilitate distal cement removal (Moreland et al. 1986, Zweymuller et al. 2005). After removing the cement, the femur is prepared to receive the revision implant. The cortical lid, which has been removed in creating the window, is replaced and secured using a cerclage wire. The femoral prosthesis can then be inserted using standard techniques. Although the risk of perforation is less with the use of a cortical window, the risk of periprosthetic fracture remains. The risk of fracture is related to the size of the window (Panjabi et al. 1985, Larson et al. 1991). Concerns about periprosthetic fractures have led to the recommendation that the cortical window should be bypassed by 2 cortical diameters, by the femoral prosthesis (Dennis et al. 1987, Larson et al. 1991, Klein and Rubash 1993). The rule of two cortical diameters is based on a finite element model by Dennis et al. (1987). There is very little biomechanical data to support this practice. Larson and associates (Larson et al. 1991) published a mechanical study on bypassing cortical defects on canine cadavers. The size of the cortical defect used in their experiment was 50% of the diaphyseal diameter, substantially larger than the window size typically used in clinical practice. The main concern with bypassing the cortical window by 2 cortical diameters is the violation of virgin bone that would otherwise be available for possible future re-revision surgery.

Zweymuller et al. (2005) reported on the use of anterior cortical windows during revision hip arthroplasty in 41 cases, where the window was not bypassed by two cortical diameters in 40 of the patients. No periprosthetic fractures were reported at an average follow-up of 7 years. These results, in addition to the present senior author's clinical experience, raised the question of the need to bypass cortical windows to prevent periprosthetic fractures. We designed a mechanical pig cadaveric study to determine and compare the risk of periprosthetic fracture for bypassed and non-bypassed anterior cortical windows.

## Materials and methods

**Figure 1.**
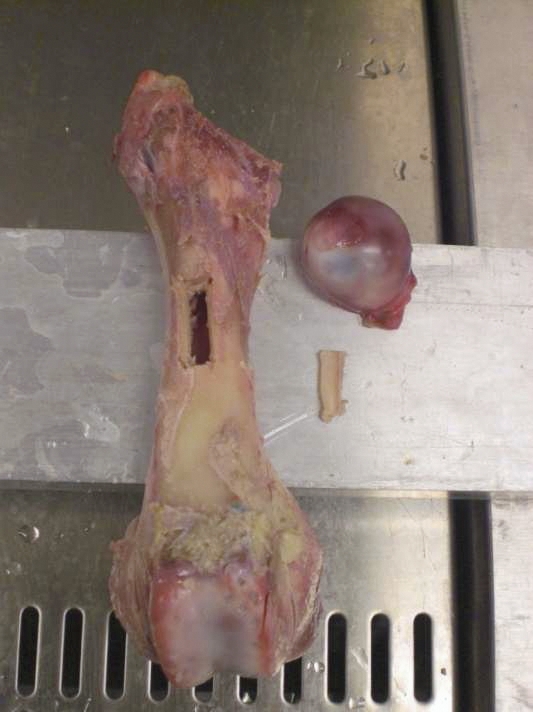
Femur prepared for implantation.

We conducted 2 separate mechanical studies on pig femurs in order to evaluate periprosthetic femoral fracture risk: 3-point bending load to failure and torsional load to failure. All specimens were dissected free of soft tissue. A custom-made cutting jig was designed to consistently create a cortical window of 10 mm × 25 mm. The size of the cortical window was based on the typical size used clinically by the senior author (RWC). The jig was positioned on the test specimens to create an anterolateral cortical window, the extent of which distally ended at the midpoint of the diaphysis (isthmus). The 4 corners of the window were predrilled using a 2-mm drill to minimize stress risers. 4 bony cuts were then made, connecting the drill holes, with an oscillating saw guided by the cutting jig. A femoral neck osteotomy was then performed, removing the femoral head (Figure 1). The femoral canal was then prepared to receive a cemented implant. A 7-mm starting drill was used to access the femoral canal. The canal was reamed with an 11-mm reamer and a 15-mm reamer. The canal was then irrigated in preparation for cementing.

A 10-mm diameter stainless steel cylindrical implant, chosen to remove the effect of implant version and relative changes in implant stiffness, was then cemented in place. Three implant tip positions, relative to the distal window edge, were investigated. The distal extent of the implant ended at the midpoint of the cortical window (–15 mm), at the distal window edge, and 15 mm beyond the distal extent of the cortical window. A bypass distance of two cortical diameters could not be used because of the prohibitive geometry of the pig femurs. The pig femurs have a relatively shorter length but similar diameter to human femurs. A bypass distance of two cortical diameters, typically 5 cm, represents approximately 10% of the length of the human femur. 15 mm was selected, as this is approximately 10% of the length of pig femurs.

All components were cemented with Paladur R50 cement. Before cementing, a distal cement plug was inserted to two cortical diameters past the distal window edge. The window was then covered with a finger while cement was applied in a retrograde manner. During pressurization of the cement, the window was sealed by hand to ensure maximal cement pressure and minimal leakage at the window. The implant was then positioned while maintaining the seal of the window. Next, the cement was trimmed from the window edges while still in a dough-like state. Finally, the window was replaced and secured with cerclage wire, ensuring that there was no cement between the edges of the window. Once the cement had hardened, the femurs were bagged as a pair and frozen at –20°C. The femurs were removed from the freezer 24 h before mechanical testing.

### 3-point bending analysis

27 pairs of fresh femurs were used. A femur of the pair was prepared at one of the implant positions and the contralateral femur was left intact to act as the control, and to allow normalization of the applied loads. 13 pairs were prepared with the implant tip at the –15-mm position, and 7 pairs were prepared at the other 2 implant locations. The larger sample size was chosen to allow us to investigate the spread in data for sizing of a clinical trial.

**Figure 2.**
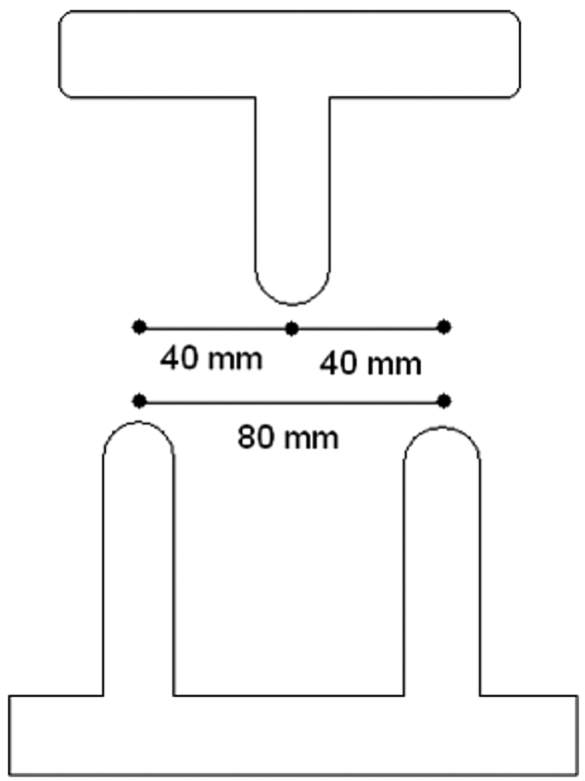
The 3-point bending setup.

**Figure 3.**
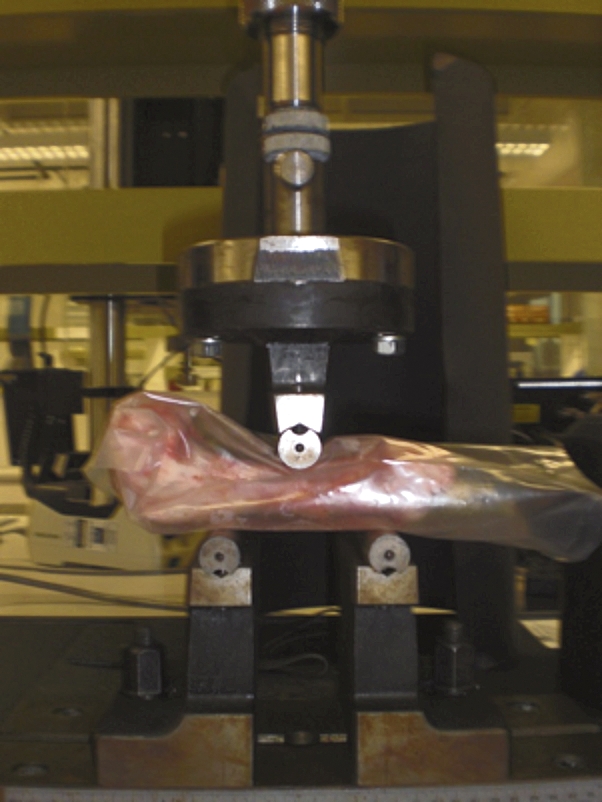
The Instron 5567 machine with femur load for 3-point bending.

All specimens were tested in a 3-point bending setup with each of the three points separated by 40 mm (Figures 2 and 3). The femur was positioned with the anterolateral aspect facing down, so that the load would be applied to the posteromedial side, opposite the cortical window, at the isthmus of the bone. An Instron 5567 machine performed the test with a strain rate of 4 mm/min. The load vs. displacement curves and maximum loads to failure were measured for all femurs. The recorded maximum load and calculated bending stiffness load for each test specimen were then divided by the value for the corresponding contralateral intact femur. This ratio indicates the relative change in strength of the femur following cortical windowing and steel-rod implantation. In addition, normalization of the test specimens with the intact femur reduces the potential confounding effect of other variables such as variation in cadaveric age, bony geometry, and bone mineral density.

### Torsional analysis

Control specimens were not used for this test. 12 matched-pair femurs (right and left) were tested to compare –15-mm and 15-mm bypass implantation. A –15-mm positioned component was randomly implanted in either the left or right femur, with the remaining contralateral femur implanted with a 15-mm bypass component as described above.

**Figure 4.**
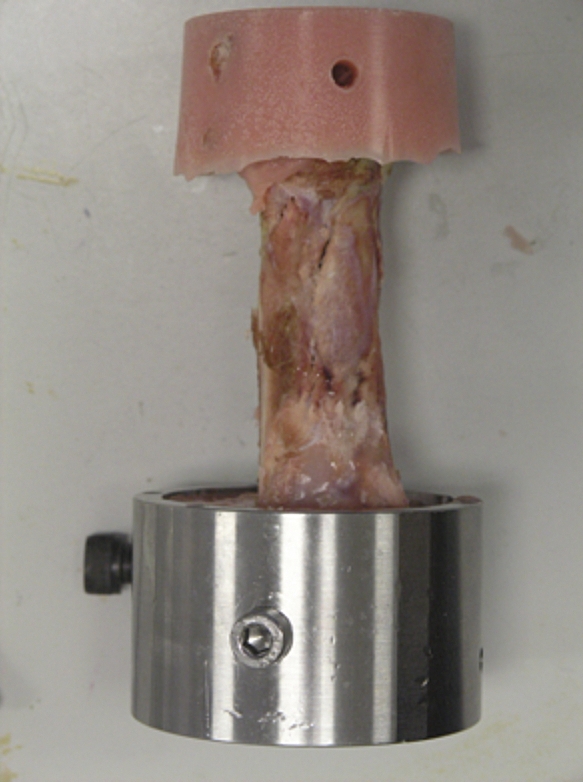
Femur prepared for torsional testing. The top cup has been removed to show the cemented ends.

Each pair of femurs was distally and proximally potted to a depth of approximately 40 mm using fixative screws and cement (Figure 4). 4 long screws were initially placed completely through both sides of the cortical bone in the distal 40 mm of the femurs. The distal end of the femur was then potted in bone cement encasing the 4 screws in order to provide torsional stability. Similarly, 4 screws were inserted in the proximal 40 mm of the femur, passing though the cortical bone and intramedullary cement while avoiding the implant. The proximal end was then potted in cement. Each specimen was loaded into an Instron 8874 machine. A gradually increasing torque was applied at a rate of 5 Nm/s until failure. The direction of the applied torque was an internal rotation torque applied proximally, as is typical during normal hip function.

### Data analysis

We examined the differences between series with analysis of variance (ANOVA) using SPSS software version 16. If p was less than 0.05, the differences between series were considered to be statistically significant. All data in graphs are presented as mean (SD).

## Results

### 3-point bending analysis

We found no statistically significant difference when comparing the maximum load to failure in any of the implant tip positions. There was a trend of reduced force required to fracture the femur as the tip of the implant moved distally (Table 1). The intact femurs broke at 4.8 kN (SD 0.7).

**Table 1.** Load to fracture ratio for the 3 implant tip positions

Test Groups (n)	Mean % (SD)	SE
–15 mm (13)	81 (14)	3.9
0 mm (7)	77 (12)	4.4
15 mm (7)	69 (17)	6.4

–15 mm vs. 15 mm, p = 0.1; –15 mm vs. 0 mm, p = 0.6; 0 mm vs. 15 mm, p = 0.3.

There was no statistically significant difference when comparing the bending stiffness ratio in any of the implant tip positions. However, the bending stiffness appeared to increase as the tip of the implant moved distally (Table 2).

**Table 2:** Bending stiffness ratio between femur pairs for the implant tip positions

Test Groups (n)	Mean % (SD)	SE
–15 mm (13)	92 (11)	2.9
0 mm (7)	87 (16)	6.0
15 mm (7)	112 (40)	15

15 mm vs. 15 mm, p = 0.1; 15 mm vs. 0 mm, p = 0.4; 0 mm vs. 15 mm, p = 0.1.

### Torsional analysis

The torque to failure of the test samples with the implant tip located at the middle of the window was 37 (SD 2.0) Nm and 37 (SD 3.5) Nm for the samples with the implant tip 15 mm past the window edge. The maximum torque to failure values for the no-bypass and bypass groups were similar with a mean of the difference between fracture torques of 0.13 (SD 2.3) Nm. In addition, there was no clear pattern between the implant position and the fracture torque; half of the samples fractured at a lower torque when the tip of the implant was located at the middle of the window.

## Discussion

Our findings suggest that bypass of an anterior cortical bone window with a long stem component during revision femoral hip replacement does not reduce the risk of postoperative periprosthetic femur fracture. This finding contradicts the conclusion drawn by Larson et al. (1991) who performed a similar mechanical study in dogs. The variation in the results is most likely related to the size of the cortical window used in the studies. Larson et al. used a 50% cortical window, which was substantially larger than the 10 mm × 25 mm window used in our study. The 10 mm × 25 mm window was deemed to be large enough to allow easy and safe removal of well-fixed cement. Although our results contradict several published clinical studies (Dennis et al. 1987, Klein and Rubash 1993, Mann et al. 1997), they is in keeping with the conclusions from a clinical paper by Zweymuller et al. (2005). The avoidance of a long-stemmed component has several advantages including cost, ease of future revision surgery, and avoidance of violation of virgin bone that might be needed in future procedures (Speirs et al. 2007).

An unexpected result that warrants discussion was the notable trend towards an increase maximum load ratio during 3-point bending for the –15-mm group compared to the 15-mm group. A possible explanation is that the distal end of the steel rod implants (theoretical stress riser) for the no bypass group specimens ends at the isthmus, where the femoral cortex is both thickest and strongest. For the bypass group, the distal end of the implant is placed more distally, at the diaphyseal-metaphyseal junction, where the femoral cortex is thin and weak. The placement of a stress riser in an area of weaker bone may play an important role in postoperative periprosthetic fracture risk. We did evaluate the possibility of placing the stress risers in the diaphyseal-metaphyseal junction but the porcine femora were too short. It is the authors opinion, given that the importance of bone morphology and geometry, that further work would be best evaluated using human cadaveric femurs.

A limitation of our study was the use of pig femurs rather that human femurs. The cortical bone of pigs and humans has been shown to have comparable mechanical properties (Aerssens et al. 1998), but the difference in bone morphology may have influenced the results.

The technique described here provides a method for removal of distal cement and one possible revision option. Long uncemented femoral revision might be a further option. However, in the case of uncemented revision the only option is to bypass the window as the location of the defect is not stabilized with a cement column. One of the advantages of revision with a cemented component, using this technique, is that the defect does not need to be bypassed with the implant, as such a standard implant could be used. In our previous work, we found that cement was suitable for stabilization of a transverse diaphyseal osteotomy, restoring 20% of the fracture load capacity (Lewis et al. 2009). It is important to consider that this technique has only been tested using cemented revisions where the cement column has the role of stabilizing the implant-bone construct.

In this work, the testing was done with healthy thick cortices, a situation that is not always found in femoral revision cases. Thus, it is important to consider that this technique is not suitable in all revision situations.

In cases of femoral revision, it is common to observe femora with compromised bone stock (Australian Orthopaedic Association 2011). In these cases, care is taken to preserve existing bone and to minimize any osteotomies during the revision procedure. The windowing technique could be applied in cases of distal lytic lesions in the cortex. Windows could be cut in or around a lesion, creating new surfaces for future healing and minimizing removal of existing bone stock.

The loading protocols we used simulate the most extreme loading conditions in the weakest directions of the bone. These directions are the tensile and shear planes. Typical forces experienced in activities of daily living are not monodirectional as tested, but a combination of forces. In most of these cases, the additional forces (compression) may help to protect the femur but this would require further studies. Another important consideration would be to investigate loading of the femur at the tip of the implant in all situations. Change of the loading in this way will mitigate any effects of the implant tip protruding past the loading point. In addition to 3-point bending, 4-point bending where the internal points are spread around the window and implant tip locations may provide further insight into the optimum location of the implant tip.

The descriptive statistics on ratio to fracture in Tables 1 and 2 show large standard deviations in all groups. Appropriate sizing of future studies will be important. The experimental variance we measured has shown that to measure a difference in mean fracture ratio of 1% (with 95% confidence) in each of the groups, –15 mm, 0 mm, and 15 mm would require 315, 240, and 560 samples, respectively. In addition, to measure a difference in bending stiffness ratio of 1% (with 95% confidence) in each of the groups would require 130, 340, and 1,275 samples.

Our study raises questions about the standard practice of bypassing cortical windows with long-stemmed cemented femoral components during revision femoral replacement. Further human cadaveric studies together with ongoing clinical evaluation are required to determine whether this technique is still warranted.
